# *catena*-Poly[[[diaqua­(nitrato-κ^2^ *O*,*O*′)(2,2′:6′,2′′-terpyridine-κ^3^ *N*,*N*′,*N*′′)neodymium(III)]-μ-cyanido-κ^2^ *N*:*C*-[dicyanidoplatinum(II)]-μ-cyanido-κ^2^ *C*:*N*] acetonitrile solvate 2,2′:6′,2′′-terpyridine hemisolvate]

**DOI:** 10.1107/S160053680903325X

**Published:** 2009-08-26

**Authors:** Branson A. Maynard, Philip A. Smith, Richard E. Sykora

**Affiliations:** a Department of Chemistry, University of South Alabama, Mobile, AL 36688-0002, USA

## Abstract

The title compound, {[NdPt(CN)_4_(NO_3_)(C_15_H_11_N_3_)(H_2_O)_2_]·CH_3_CN·0.5C_15_H_11_N_3_}_*n*_, was isolated from solution as a one-dimensional coordination polymer. The Nd^3+^ site in the structure has a ninefold coordination with a distorted tricapped trigonal-prismatic geometry, while the Pt^II^ ion is coordinated by four cyanide groups in an almost regular square-planar geometry. *Cis*-bridging by the tetracyanidoplatinate anions links the Nd^3+^ cations, forming the one-dimensional chains. Additionally, each Nd^3+^ contains coordin­ation by two water mol­ecules, one tridentate 2,2′:6′,2′′-terpyridine mol­ecule, and one bidentate nitrate anion. 2,2′:6′,2′′-Terpyridine and acetonitrile solvent mol­ecules are incorporated between the chains, the former form π-stacking inter­actions (average inter­planar distance 3.33 Å) with terpyridine mol­ecules located in the chains. Relatively long Pt⋯Pt inter­actions [3.847 (1) Å] are observed in the structure. O—H⋯N and O—H⋯O hydrogen bonding interactions between the consituents consolidates the crystal packing.

## Related literature

For related lanthanide tetracyanidoplatinate structures containing 2,2′:6′,2′′-terpyridine, see: Maynard *et al.* (2008 ▶); Maynard, Smith, Ladner *et al.* (2009 ▶); Maynard, Smith, Jaleel *et al.* (2009 ▶). For structural and spectroscopic information on simpler lanthanide tetracyanidoplatinates, see: Gliemann & Yersin (1985 ▶); Holzapfel *et al.* (1981 ▶). For luminescence data on lanthanide terpyridine systems, see: Mukkala *et al.* (1995 ▶). 
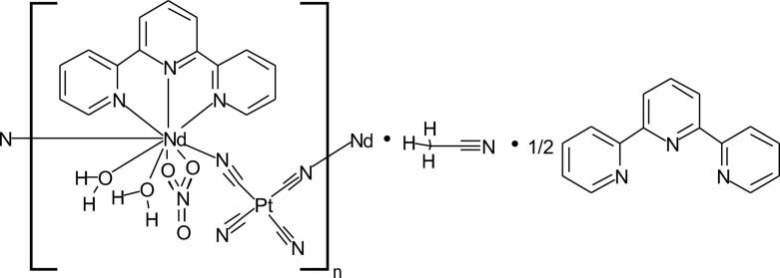


## Experimental

### 

#### Crystal data

[NdPt(CN)_4_(NO_3_)(C_15_H_11_N_3_)(H_2_O)_2_]·C_2_H_3_N·0.5C_15_H_11_N_3_
*M* *_r_* = 932.4
Monoclinic, 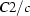

*a* = 33.231 (6) Å
*b* = 14.3642 (17) Å
*c* = 13.823 (3) Å
β = 108.931 (16)°
*V* = 6241.5 (19) Å^3^
*Z* = 8
Mo *K*α radiation
μ = 6.18 mm^−1^
*T* = 290 K
0.45 × 0.17 × 0.08 mm

#### Data collection

Enraf–Nonius CAD-4 diffractometer
Absorption correction: analytical (*XPREP*; Bruker, 1998 ▶) *T*_min_ = 0.308, *T*_max_ = 0.632
5824 measured reflections
5722 independent reflections
4089 reflections with *I* > 2σ(*I*)
*R*_int_ = 0.031
3 standard reflections frequency: 120 min intensity decay: none

#### Refinement

*R*[*F* ^2^ > 2σ(*F* ^2^)] = 0.036
*wR*(*F* ^2^) = 0.087
*S* = 1.00
5722 reflections
420 parameters
H-atom parameters constrained
Δρ_max_ = 0.86 e Å^−3^
Δρ_min_ = −0.84 e Å^−3^

### 

Data collection: *CAD-4-PC Software* (Enraf–Nonius, 1993 ▶); cell refinement: *CAD-4-PC Software*; data reduction: *XCAD4* (Harms & Wocadlo, 1996 ▶); program(s) used to solve structure: *SHELXS97* (Sheldrick, 2008 ▶); program(s) used to refine structure: *SHELXL97* (Sheldrick, 2008 ▶); molecular graphics: *SHELXTL* (Sheldrick, 2008 ▶); software used to prepare material for publication: *publCIF* (Westrip, 2009 ▶).

## Supplementary Material

Crystal structure: contains datablocks I, global. DOI: 10.1107/S160053680903325X/nc2153sup1.cif

Structure factors: contains datablocks I. DOI: 10.1107/S160053680903325X/nc2153Isup2.hkl

Additional supplementary materials:  crystallographic information; 3D view; checkCIF report

## Figures and Tables

**Table 1:** Hydrogen-bond geometry (Å, °)

*D*—H⋯*A*	*D*—H	H⋯*A*	*D*⋯*A*	*D*—H⋯*A*
O4—H4*A*⋯N4^i^	0.85	2.00	2.760 (9)	149.1
O4—H4*B*⋯N3^ii^	0.85	2.00	2.814 (10)	160.5
O5—H5*B*⋯N9^iii^	0.85	2.16	2.993 (9)	167.4
O5—H5*C*⋯O1^iv^	0.85	1.99	2.770 (8)	152.2

Symmetry codes: (i) 
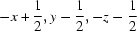
; (ii) 
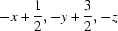
; (iii) 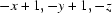; (iv) 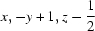.

**Fig. 1.**
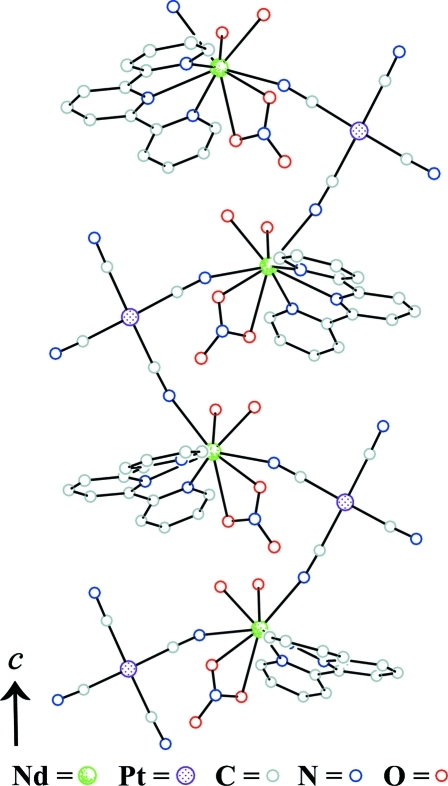
A representation of the one-dimensional chains that extend along the c axis in (I).

**Fig. 2.**
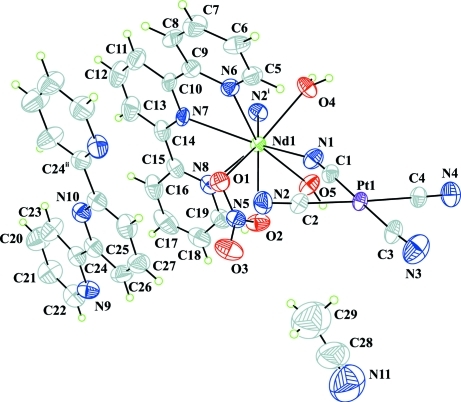
A thermal ellipsoid plot of (I) with the atom-numbering scheme. Displacement ellipsoids for non-hydrogen atoms are drawn at the 50% probability level. H-atoms are shown as spheres of arbitrary size. Symmetry codes: (i) x, -y + 1, z - 1/2; (ii) -x + 1, y, -z + 1/2.

**Fig. 3.**
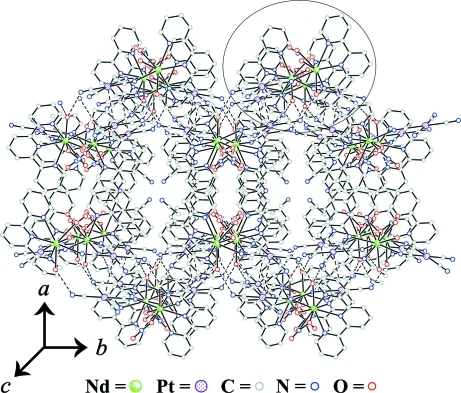
A packing diagram for (I) viewed along the c axis, the direction parallel to the 1-D chains. Pt—Pt and hydrogen-bonding interactions are shown by the dashed lines and one of the 1-D chains is circled for clarity.

## supplementary crystallographic information

### Comment

One of our research goals is to prepare systems where the generally weak Ln^3+^ emissions are enhanced through the use of sensitizing ligands coordinated directly to Ln^3+^ cations. Recent efforts in our lab have focused on the novel lanthanide compounds that incorporate two ligand groups simultaneously to achieve this goal. The effort has focused on preparing lanthanide compounds that contain both tetracyanoplatinate(II) anions (TCP) and 2,2':6',2''-terpyridine (tpy) ligands, since each of these ligands have been shown to act as sensitizers for various Ln^3+^ cations (Gliemann & Yersin, 1985; Mukkala *et al.*, 1995). We recently communicated some of our findings in this area (Maynard *et al.*, 2008; Maynard, Smith, Ladner *et al.*, 2009). Through our efforts we have prepared a number of novel compounds incorporating various Ln^3+^ cations, terpyridine, and TCP anions and have also recently reported on these structures (Maynard *et al.*, 2008; Maynard, Smith, Ladner *et al.*, 2009; Maynard, Smith, Jaleel, *et al.*, 2009).

The title compound, (I), is similar to several previously reported compounds in that it contains one-dimensional [Nd(C_15_H_11_N_3_)(H_2_O)_2_(NO_3_)(Pt(CN)_4_)] chains reminiscent of those found in Ln(C_15_H_11_N_3_)(H_2_O)_2_(NO_3_)[Pt(CN)_4_].CH_3_CN (Ln = Eu (Maynard *et al.*, 2008; Maynard, Smith, Ladner *et al.*, 2009) or Ln = Ho (Maynard, Smith, Jaleel, *et al.*, 2009)) and Yb(C_15_H_11_N_3_)(H_2_O)_2_(NO_3_)[Pt(CN)_4_].0.5CH_3_CN.1.5H_2_O (Maynard, Smith, Jaleel, *et al.*, 2009). The major structural differences between these related structure types can be attributed in part to the crystallization of various solvent or guest molecules between the one-dimensional chains.

The neutral, one-dimensional [Nd(C_15_H_11_N_3_)(H_2_O)_2_(NO_3_)(Pt(CN)_4_)] chains in the structure of (I) are illustrated in Figure 1 and a thermal ellipsoid plot of the asymmetric unit is illustrated in Figure 2. The chains are formed by the linkage of the Nd^3+^ cations by *cis*-bridging tetracyanoplatinate anions. The coordination of the Nd site is ninefold and can be described as a distorted [NdO_4_N_5_] tri-capped trigonal prism. The five nitrogen atoms in the inner sphere of the Nd^3+^ cations result from the coordination of one tridentate terpyridine ligand and two N-bound TCP anions while the four oxygen atoms are a result of one bidentate nitrate anion and two coordinated water molecules. The two longest Nd—O bond distances for each compound are those to the nitrate anion. The Nd—N bonds to the cyano groups are shorter by an average of ~0.08 Å than the Nd—N bonds to the tpy molecule. The Pt—C distances have an average of 1.984 (8) Å.

The packing diagram of (I) viewed along the *c* axis is shown in Figure 3. The predominant inter-chain feature is the existence of Pt—Pt interactions. These interactions in (I) are quite long (3.847 (1) Å), but are otherwise reminiscent of those observed in earlier reported lanthanide TCP compounds in that they form dimeric groups (Maynard, Smith, Ladner *et al.*, 2009; Maynard, Smith, Jaleel, *et al.*, 2009). This is in contrast to many reported lanthanide TCP compounds where there exist pseudo-1-D columnar stacks (Gliemann & Yersin, 1985; Holzapfel *et al.*, 1981) containing planar TCP anions parallel to one another. Additional features found in the packing diagram for (I) include porous channels along the *c* axis that contain acetonitrile solvate molecules, numerous inter-chain hydrogen bonding interactions, and also the presence of π-stacking interactions. These latter interactions (3.33 Å average distance between planes) are between the coordinated tpy and the guest tpy molecule that is co-crystallized between the one-dimensional chains. Also worth noting is the orientation of the coordinated tpy molecules in the one-dimensional chains; viewing along the *c* axis reveals that these molecules are located on either side of the chains. A similar situation also occurs in Eu(C_15_H_11_N_3_)(H_2_O)_2_(NO_3_)[Pt(CN)_4_].CH_3_CN (Maynard *et al.*, 2008; Maynard, Smith, Ladner *et al.*, 2009) while Yb(C_15_H_11_N_3_)(H_2_O)_2_(NO_3_)[Pt(CN)_4_].0.5CH_3_CN.1.5H_2_O (Maynard, Smith, Jaleel, *et al.*, 2009) contains one-dimensional chains where all of the terpyridine molecules reside on a single side of the chain.

### Experimental

The title compound was synthesized by reacting Nd(NO_3_).6H_2_O (Strem, 99.9%), K_2_Pt(CN)_4_.3H_2_O (Alfa Aesar, 99.9%), and 2,2':6',2''-terpyridine (Aldrich, 98%) in a 1:1:1 molar ratio. The reaction proceeded by adding 1 ml of a 0.10 *M* solution of potassium tetracyanoplatinate in 20%:80% water:acetonitrile mixture to 1 ml of a 0.10 *M* solution of neodymium nitrate in acetonitrile. Next, 1 ml of a 0.10 *M* solution of 2,2':6',2''-terpyridine in acetonitrile was layered on the former mixture. Purple crystals were harvested from the reaction tube after several days.

### Refinement

Hydrogen atoms on the terpyridine rings and acetonitrile molecule were placed in calculated positions (the acetonitrile H atoms were allowed to rotate but not to tip) and allowed to ride during subsequent refinement, with *U*_iso_(H) = 1.2*U*_eq_(C) and C—H distances of 0.93 Å for the former and *U*_iso_(H) = 1.5*U*_eq_(C) and C—H distances of 0.96 Å for the latter. H-atoms contained in the water molecules were initially located in the difference map and then constrained to have O—H distances of 0.85 Å and *U*_iso_(H) = 1.2*U*_eq_(O).

### Crystal data

**Table:**

[NdPt(CN)_4_(NO_3_)(C_15_H_11_N_3_)(H_2_O)_2_]·C_2_H_3_N·0.5C_15_H_11_N_3_	*F*(000) = 3568
*M**_r_* = 932.4	*D*_x_ = 1.985 Mg m^−^^3^
Monoclinic, *C*2/*c*	Mo *K*α radiation, λ = 0.71073 Å
Hall symbol: -C 2yc	Cell parameters from 25 reflections
*a* = 33.231 (6) Å	θ = 8.5–15.4°
*b* = 14.3642 (17) Å	µ = 6.18 mm^−^^1^
*c* = 13.823 (3) Å	*T* = 290 K
β = 108.931 (16)°	Plate, purple
*V* = 6241.5 (19) Å^3^	0.45 × 0.17 × 0.08 mm
*Z* = 8	

### Data collection

**Table:**

Enraf–Nonius CAD-4 diffractometer	4089 reflections with *I* > 2σ(*I*)
Radiation source: fine-focus sealed tube	*R*_int_ = 0.031
graphite	θ_max_ = 25.4°, θ_min_ = 2.1°
θ/2θ scans	*h* = 0→40
Absorption correction: analytical (*SADABS*; Bruker, 1998)	*k* = 0→17
*T*_min_ = 0.308, *T*_max_ = 0.632	*l* = −16→15
5824 measured reflections	3 standard reflections every 120 min
5722 independent reflections	intensity decay: none

### Refinement

**Table:**

Refinement on *F*^2^	Primary atom site location: structure-invariant direct methods
Least-squares matrix: full	Secondary atom site location: difference Fourier map
*R*[*F*^2^ > 2σ(*F*^2^)] = 0.036	Hydrogen site location: mixed
*wR*(*F*^2^) = 0.087	H-atom parameters constrained
*S* = 1.00	*w* = 1/[σ^2^(*F*_o_^2^) + (0.0283*P*)^2^] where *P* = (*F*_o_^2^ + 2*F*_c_^2^)/3
5722 reflections	(Δ/σ)_max_ = 0.001
420 parameters	Δρ_max_ = 0.86 e Å^−^^3^
0 restraints	Δρ_min_ = −0.84 e Å^−^^3^
44 constraints	

### Special details

**Table:**

Geometry. All e.s.d.'s (except the e.s.d. in the dihedral angle between two l.s. planes) are estimated using the full covariance matrix. The cell e.s.d.'s are taken into account individually in the estimation of e.s.d.'s in distances, angles and torsion angles; correlations between e.s.d.'s in cell parameters are only used when they are defined by crystal symmetry. An approximate (isotropic) treatment of cell e.s.d.'s is used for estimating e.s.d.'s involving l.s. planes.
Refinement. Refinement of *F*^2^ against ALL reflections. The weighted *R*-factor *wR* and goodness of fit *S* are based on *F*^2^, conventional *R*-factors *R* are based on *F*, with *F* set to zero for negative *F*^2^. The threshold expression of *F*^2^ > σ(*F*^2^) is used only for calculating *R*-factors(gt) *etc*. and is not relevant to the choice of reflections for refinement. *R*-factors based on *F*^2^ are statistically about twice as large as those based on *F*, and *R*- factors based on ALL data will be even larger.

### Fractional atomic coordinates and isotropic or equivalent isotropic displacement parameters (Å^2^)

**Table:**

	*x*	*y*	*z*	*U*_iso_*/*U*_eq_	
Nd1	0.349783 (14)	0.43585 (3)	−0.06095 (3)	0.02261 (11)	
Pt1	0.308541 (10)	0.775186 (19)	0.07657 (2)	0.02355 (9)	
C1	0.3149 (3)	0.6590 (6)	0.0072 (6)	0.0287 (18)	
C2	0.3302 (3)	0.7125 (5)	0.2122 (6)	0.0277 (17)	
C3	0.3023 (3)	0.8936 (6)	0.1443 (6)	0.0330 (19)	
C4	0.2851 (3)	0.8356 (5)	−0.0596 (6)	0.0302 (18)	
C5	0.2743 (3)	0.4274 (6)	0.0670 (7)	0.039 (2)	
H5A	0.2704	0.4877	0.0410	0.046*	
C6	0.2505 (3)	0.3991 (6)	0.1269 (7)	0.044 (2)	
H6A	0.2307	0.4391	0.1391	0.053*	
C7	0.2562 (3)	0.3117 (7)	0.1679 (7)	0.052 (3)	
H7A	0.2411	0.2918	0.2102	0.062*	
C8	0.2849 (3)	0.2537 (7)	0.1451 (7)	0.042 (2)	
H8A	0.2891	0.1934	0.1709	0.051*	
C9	0.3075 (3)	0.2860 (5)	0.0832 (6)	0.0329 (19)	
C10	0.3394 (3)	0.2280 (6)	0.0592 (6)	0.035 (2)	
C11	0.3405 (3)	0.1306 (7)	0.0718 (7)	0.050 (3)	
H11A	0.3202	0.1003	0.0936	0.060*	
C12	0.3719 (4)	0.0820 (7)	0.0512 (9)	0.068 (3)	
H12A	0.3729	0.0176	0.0582	0.082*	
C13	0.4015 (4)	0.1255 (7)	0.0211 (8)	0.057 (3)	
H13A	0.4231	0.0914	0.0087	0.069*	
C14	0.4000 (3)	0.2214 (6)	0.0084 (7)	0.039 (2)	
C15	0.4318 (3)	0.2724 (6)	−0.0214 (6)	0.035 (2)	
C16	0.4697 (3)	0.2336 (8)	−0.0204 (8)	0.056 (3)	
H16A	0.4750	0.1711	−0.0036	0.067*	
C17	0.4999 (3)	0.2866 (10)	−0.0443 (9)	0.069 (4)	
H17A	0.5255	0.2604	−0.0442	0.083*	
C18	0.4915 (3)	0.3766 (9)	−0.0677 (7)	0.058 (3)	
H18A	0.5112	0.4140	−0.0841	0.069*	
C19	0.4530 (3)	0.4133 (7)	−0.0669 (7)	0.045 (2)	
H19A	0.4472	0.4757	−0.0837	0.054*	
C20	0.5895 (4)	0.0801 (8)	0.1679 (10)	0.073 (4)	
H20A	0.5883	0.0154	0.1653	0.088*	
C21	0.6217 (3)	0.1261 (8)	0.1448 (8)	0.060 (3)	
H21A	0.6423	0.0937	0.1265	0.072*	
C22	0.6220 (3)	0.2221 (8)	0.1500 (8)	0.055 (3)	
H22A	0.6435	0.2536	0.1340	0.066*	
C23	0.5593 (3)	0.1288 (7)	0.1946 (8)	0.056 (3)	
H23A	0.5373	0.0984	0.2094	0.068*	
C24	0.5627 (3)	0.2254 (6)	0.1988 (6)	0.039 (2)	
C25	0.5303 (3)	0.2810 (6)	0.2270 (6)	0.038 (2)	
C26	0.5314 (3)	0.3784 (7)	0.2262 (7)	0.048 (2)	
H26A	0.5527	0.4102	0.2100	0.058*	
C27	0.5000	0.4252 (10)	0.2500	0.053 (4)	
H27A	0.5000	0.4899	0.2500	0.063*	
C28	0.4505 (6)	0.8941 (11)	0.1457 (17)	0.109 (7)	
C29	0.4263 (8)	0.8338 (13)	0.1910 (18)	0.134 (7)	
H29A	0.3967	0.8499	0.1644	0.4 (2)*	
H29B	0.4362	0.8416	0.2639	0.12 (7)*	
H29C	0.4300	0.7701	0.1745	0.09 (4)*	
N1	0.3189 (2)	0.5906 (5)	−0.0313 (5)	0.0376 (17)	
N2	0.3413 (2)	0.6737 (5)	0.2886 (5)	0.0378 (18)	
N3	0.2968 (3)	0.9628 (5)	0.1791 (7)	0.055 (2)	
N4	0.2716 (3)	0.8690 (5)	−0.1384 (6)	0.046 (2)	
N5	0.4061 (2)	0.5440 (5)	0.1161 (5)	0.0326 (16)	
N6	0.3027 (2)	0.3734 (5)	0.0445 (5)	0.0327 (16)	
N7	0.3677 (2)	0.2706 (4)	0.0232 (5)	0.0304 (15)	
N8	0.4242 (2)	0.3633 (5)	−0.0434 (5)	0.0358 (17)	
N9	0.5936 (2)	0.2724 (5)	0.1765 (6)	0.0447 (19)	
N10	0.5000	0.2344 (7)	0.2500	0.039 (2)	
N11	0.4708 (5)	0.9406 (10)	0.1179 (14)	0.130 (6)	
O1	0.38212 (18)	0.4789 (4)	0.1277 (4)	0.0366 (14)	
O2	0.40884 (19)	0.5548 (4)	0.0286 (4)	0.0418 (15)	
O3	0.4259 (2)	0.5928 (5)	0.1889 (5)	0.058 (2)	
O4	0.27728 (18)	0.4401 (4)	−0.1750 (4)	0.0374 (14)	
H4A	0.2716	0.4145	−0.2333	0.045*	
H4B	0.2556	0.4753	−0.1890	0.045*	
O5	0.3686 (2)	0.5374 (4)	−0.1855 (4)	0.0402 (15)	
H5B	0.3769	0.5937	−0.1773	0.048*	
H5C	0.3765	0.5152	−0.2334	0.048*	

### Atomic displacement parameters (Å^2^)

**Table:**

	*U*^11^	*U*^22^	*U*^33^	*U*^12^	*U*^13^	*U*^23^
Nd1	0.0283 (2)	0.0236 (2)	0.0168 (2)	−0.00194 (17)	0.00833 (17)	−0.00252 (16)
Pt1	0.03025 (17)	0.02168 (15)	0.02051 (15)	0.00316 (14)	0.01069 (12)	0.00322 (12)
C1	0.032 (5)	0.031 (4)	0.027 (4)	0.000 (4)	0.015 (4)	0.007 (4)
C2	0.037 (5)	0.024 (4)	0.025 (4)	0.001 (4)	0.015 (4)	−0.001 (3)
C3	0.048 (5)	0.027 (4)	0.027 (4)	0.005 (4)	0.017 (4)	0.005 (4)
C4	0.036 (5)	0.030 (4)	0.027 (4)	0.005 (4)	0.013 (4)	0.004 (4)
C5	0.037 (5)	0.033 (5)	0.047 (5)	−0.003 (4)	0.016 (4)	−0.001 (4)
C6	0.045 (6)	0.048 (5)	0.047 (5)	−0.017 (5)	0.026 (5)	−0.010 (5)
C7	0.055 (7)	0.071 (7)	0.035 (5)	−0.016 (6)	0.021 (5)	0.010 (5)
C8	0.036 (5)	0.051 (5)	0.050 (6)	−0.006 (4)	0.027 (5)	0.020 (5)
C9	0.039 (5)	0.034 (4)	0.025 (4)	−0.006 (4)	0.009 (4)	−0.005 (4)
C10	0.043 (5)	0.038 (5)	0.019 (4)	−0.005 (4)	0.004 (4)	0.010 (4)
C11	0.058 (7)	0.043 (6)	0.052 (6)	−0.001 (5)	0.021 (5)	0.013 (5)
C12	0.081 (9)	0.029 (5)	0.090 (9)	0.016 (6)	0.021 (7)	0.015 (5)
C13	0.063 (7)	0.045 (6)	0.067 (7)	0.019 (5)	0.025 (6)	0.009 (5)
C14	0.039 (5)	0.041 (5)	0.037 (5)	0.013 (4)	0.012 (4)	0.003 (4)
C15	0.036 (5)	0.048 (5)	0.020 (4)	0.007 (4)	0.007 (4)	−0.004 (4)
C16	0.050 (6)	0.065 (7)	0.053 (6)	0.024 (6)	0.019 (5)	0.010 (5)
C17	0.032 (6)	0.116 (11)	0.062 (7)	0.017 (7)	0.018 (5)	−0.004 (8)
C18	0.044 (6)	0.095 (9)	0.032 (5)	−0.015 (6)	0.012 (5)	0.008 (6)
C19	0.034 (5)	0.064 (6)	0.038 (5)	−0.001 (5)	0.015 (4)	0.002 (5)
C20	0.076 (9)	0.050 (6)	0.105 (10)	0.005 (6)	0.047 (8)	−0.004 (7)
C21	0.060 (7)	0.068 (7)	0.069 (7)	0.007 (6)	0.045 (6)	−0.008 (6)
C22	0.055 (7)	0.071 (7)	0.047 (6)	0.004 (6)	0.026 (5)	−0.004 (6)
C23	0.067 (7)	0.041 (5)	0.077 (8)	0.007 (5)	0.046 (6)	0.004 (5)
C24	0.038 (5)	0.050 (5)	0.031 (5)	0.005 (5)	0.014 (4)	−0.001 (4)
C25	0.036 (5)	0.057 (6)	0.024 (4)	0.002 (5)	0.014 (4)	0.000 (4)
C26	0.046 (6)	0.050 (6)	0.050 (6)	−0.016 (5)	0.018 (5)	0.000 (5)
C27	0.039 (8)	0.042 (8)	0.074 (11)	0.000	0.016 (8)	0.000
C28	0.080 (12)	0.061 (10)	0.17 (2)	−0.003 (8)	0.019 (12)	−0.024 (11)
C29	0.14 (2)	0.103 (15)	0.16 (2)	−0.002 (13)	0.052 (16)	−0.022 (13)
N1	0.048 (5)	0.030 (4)	0.038 (4)	−0.002 (3)	0.018 (4)	−0.003 (3)
N2	0.040 (5)	0.040 (4)	0.031 (4)	0.003 (3)	0.009 (3)	0.008 (3)
N3	0.070 (6)	0.036 (4)	0.070 (6)	0.008 (4)	0.039 (5)	−0.004 (4)
N4	0.052 (5)	0.048 (5)	0.036 (4)	0.008 (4)	0.011 (4)	0.007 (4)
N5	0.031 (4)	0.038 (4)	0.028 (4)	−0.002 (3)	0.009 (3)	−0.004 (3)
N6	0.036 (4)	0.032 (4)	0.029 (4)	−0.005 (3)	0.008 (3)	−0.001 (3)
N7	0.039 (4)	0.028 (3)	0.022 (3)	0.001 (3)	0.008 (3)	0.008 (3)
N8	0.036 (4)	0.049 (4)	0.024 (4)	0.001 (4)	0.012 (3)	0.001 (3)
N9	0.041 (5)	0.056 (5)	0.043 (4)	−0.008 (4)	0.020 (4)	−0.008 (4)
N10	0.040 (6)	0.047 (6)	0.031 (5)	0.000	0.012 (5)	0.000
N11	0.122 (14)	0.102 (12)	0.173 (16)	0.015 (10)	0.060 (12)	−0.002 (11)
O1	0.043 (4)	0.041 (3)	0.024 (3)	−0.005 (3)	0.010 (3)	−0.002 (3)
O2	0.046 (4)	0.051 (4)	0.033 (3)	−0.018 (3)	0.020 (3)	−0.007 (3)
O3	0.055 (5)	0.070 (5)	0.040 (4)	−0.015 (4)	0.001 (3)	−0.026 (4)
O4	0.032 (3)	0.048 (3)	0.027 (3)	0.007 (3)	0.002 (3)	−0.012 (3)
O5	0.063 (4)	0.033 (3)	0.032 (3)	−0.006 (3)	0.026 (3)	−0.002 (3)

### Geometric parameters (Å, °)

**Table:**

Nd1—O4	2.414 (5)	C15—C16	1.374 (12)
Nd1—O5	2.486 (5)	C16—C17	1.383 (15)
Nd1—N1	2.536 (7)	C16—H16A	0.9300
Nd1—N2^i^	2.550 (7)	C17—C18	1.339 (16)
Nd1—O1	2.554 (5)	C17—H17A	0.9300
Nd1—O2	2.594 (6)	C18—C19	1.389 (14)
Nd1—N6	2.619 (7)	C18—H18A	0.9300
Nd1—N8	2.623 (7)	C19—N8	1.317 (11)
Nd1—N7	2.625 (6)	C19—H19A	0.9300
Nd1—N5	2.989 (7)	C20—C23	1.367 (14)
Pt1—C1	1.970 (8)	C20—C21	1.380 (14)
Pt1—C3	1.985 (8)	C20—H20A	0.9300
Pt1—C4	1.988 (8)	C21—C22	1.380 (14)
Pt1—C2	1.992 (8)	C21—H21A	0.9300
C1—N1	1.146 (10)	C22—N9	1.331 (12)
C2—N2	1.144 (9)	C22—H22A	0.9300
C3—N3	1.144 (10)	C23—C24	1.392 (12)
C4—N4	1.140 (10)	C23—H23A	0.9300
C5—N6	1.334 (11)	C24—N9	1.347 (11)
C5—C6	1.379 (12)	C24—C25	1.491 (12)
C5—H5A	0.9300	C25—N10	1.329 (10)
C6—C7	1.365 (13)	C25—C26	1.400 (12)
C6—H6A	0.9300	C26—C27	1.367 (11)
C7—C8	1.378 (14)	C26—H26A	0.9300
C7—H7A	0.9300	C27—C26^ii^	1.367 (11)
C8—C9	1.388 (11)	C27—H27A	0.9300
C8—H8A	0.9300	C28—N11	1.10 (2)
C9—N6	1.355 (10)	C28—C29	1.46 (2)
C9—C10	1.469 (12)	C29—H29A	0.9600
C10—N7	1.344 (10)	C29—H29B	0.9600
C10—C11	1.410 (12)	C29—H29C	0.9600
C11—C12	1.360 (14)	N2—Nd1^iii^	2.550 (7)
C11—H11A	0.9300	N5—O3	1.227 (8)
C12—C13	1.340 (15)	N5—O2	1.252 (8)
C12—H12A	0.9300	N5—O1	1.273 (8)
C13—C14	1.387 (13)	N10—C25^ii^	1.329 (10)
C13—H13A	0.9300	O4—H4A	0.8500
C14—N7	1.356 (10)	O4—H4B	0.8499
C14—C15	1.449 (12)	O5—H5B	0.8500
C15—N8	1.344 (11)	O5—H5C	0.8499
			
O4—Nd1—O5	87.4 (2)	C12—C13—C14	120.2 (10)
O4—Nd1—N1	73.3 (2)	C12—C13—H13A	119.9
O5—Nd1—N1	78.7 (2)	C14—C13—H13A	119.9
O4—Nd1—N2^i^	70.1 (2)	N7—C14—C13	119.7 (9)
O5—Nd1—N2^i^	77.5 (2)	N7—C14—C15	117.7 (7)
N1—Nd1—N2^i^	136.8 (2)	C13—C14—C15	122.6 (9)
O4—Nd1—O1	131.37 (19)	N8—C15—C16	119.9 (9)
O5—Nd1—O1	116.64 (18)	N8—C15—C14	117.1 (8)
N1—Nd1—O1	71.4 (2)	C16—C15—C14	122.8 (9)
N2^i^—Nd1—O1	151.8 (2)	C15—C16—C17	120.6 (10)
O4—Nd1—O2	137.3 (2)	C15—C16—H16A	119.7
O5—Nd1—O2	67.83 (18)	C17—C16—H16A	119.7
N1—Nd1—O2	68.2 (2)	C18—C17—C16	118.6 (10)
N2^i^—Nd1—O2	131.5 (2)	C18—C17—H17A	120.7
O1—Nd1—O2	49.51 (17)	C16—C17—H17A	120.7
O4—Nd1—N6	73.9 (2)	C17—C18—C19	119.0 (10)
O5—Nd1—N6	156.4 (2)	C17—C18—H18A	120.5
N1—Nd1—N6	82.2 (2)	C19—C18—H18A	120.5
N2^i^—Nd1—N6	108.3 (2)	N8—C19—C18	122.6 (10)
O1—Nd1—N6	69.15 (19)	N8—C19—H19A	118.7
O2—Nd1—N6	117.16 (19)	C18—C19—H19A	118.7
O4—Nd1—N8	141.46 (19)	C23—C20—C21	120.5 (10)
O5—Nd1—N8	81.7 (2)	C23—C20—H20A	119.7
N1—Nd1—N8	138.8 (2)	C21—C20—H20A	119.7
N2^i^—Nd1—N8	71.4 (2)	C20—C21—C22	117.4 (10)
O1—Nd1—N8	85.98 (19)	C20—C21—H21A	121.3
O2—Nd1—N8	70.9 (2)	C22—C21—H21A	121.3
N6—Nd1—N8	121.9 (2)	N9—C22—C21	124.2 (10)
O4—Nd1—N7	110.3 (2)	N9—C22—H22A	117.9
O5—Nd1—N7	140.0 (2)	C21—C22—H22A	117.9
N1—Nd1—N7	140.0 (2)	C20—C23—C24	117.8 (10)
N2^i^—Nd1—N7	75.7 (2)	C20—C23—H23A	121.1
O1—Nd1—N7	78.85 (18)	C24—C23—H23A	121.1
O2—Nd1—N7	110.9 (2)	N9—C24—C23	123.1 (9)
N6—Nd1—N7	62.3 (2)	N9—C24—C25	117.5 (8)
N8—Nd1—N7	61.8 (2)	C23—C24—C25	119.4 (9)
O4—Nd1—N5	138.48 (18)	N10—C25—C26	121.9 (9)
O5—Nd1—N5	91.78 (19)	N10—C25—C24	117.4 (8)
N1—Nd1—N5	65.9 (2)	C26—C25—C24	120.7 (8)
N2^i^—Nd1—N5	149.6 (2)	C27—C26—C25	117.7 (10)
O1—Nd1—N5	24.99 (17)	C27—C26—H26A	121.2
O2—Nd1—N5	24.64 (17)	C25—C26—H26A	121.2
N6—Nd1—N5	92.92 (19)	C26—C27—C26^ii^	121.2 (13)
N8—Nd1—N5	79.0 (2)	C26—C27—H27A	119.4
N7—Nd1—N5	96.63 (19)	C26^ii^—C27—H27A	119.4
C1—Pt1—C3	178.9 (3)	N11—C28—C29	175 (3)
C1—Pt1—C4	88.8 (3)	C28—C29—H29A	109.5
C3—Pt1—C4	90.2 (3)	C28—C29—H29B	109.5
C1—Pt1—C2	90.7 (3)	H29A—C29—H29B	109.5
C3—Pt1—C2	90.3 (3)	C28—C29—H29C	109.5
C4—Pt1—C2	178.1 (3)	H29A—C29—H29C	109.5
N1—C1—Pt1	178.6 (8)	H29B—C29—H29C	109.5
N2—C2—Pt1	177.1 (8)	C1—N1—Nd1	160.2 (7)
N3—C3—Pt1	176.4 (9)	C2—N2—Nd1^iii^	166.1 (7)
N4—C4—Pt1	179.0 (7)	O3—N5—O2	122.3 (7)
N6—C5—C6	123.8 (8)	O3—N5—O1	120.4 (7)
N6—C5—H5A	118.1	O2—N5—O1	117.3 (6)
C6—C5—H5A	118.1	O3—N5—Nd1	173.9 (6)
C7—C6—C5	119.4 (10)	O2—N5—Nd1	59.7 (4)
C7—C6—H6A	120.3	O1—N5—Nd1	58.0 (3)
C5—C6—H6A	120.3	C5—N6—C9	116.7 (8)
C6—C7—C8	118.4 (9)	C5—N6—Nd1	121.8 (5)
C6—C7—H7A	120.8	C9—N6—Nd1	121.5 (6)
C8—C7—H7A	120.8	C10—N7—C14	120.1 (7)
C7—C8—C9	119.4 (9)	C10—N7—Nd1	119.4 (5)
C7—C8—H8A	120.3	C14—N7—Nd1	118.8 (5)
C9—C8—H8A	120.3	C19—N8—C15	119.3 (8)
N6—C9—C8	122.4 (8)	C19—N8—Nd1	119.8 (6)
N6—C9—C10	116.0 (7)	C15—N8—Nd1	120.5 (6)
C8—C9—C10	121.6 (8)	C22—N9—C24	116.9 (8)
N7—C10—C11	120.4 (9)	C25—N10—C25^ii^	119.6 (11)
N7—C10—C9	117.9 (7)	N5—O1—Nd1	97.0 (4)
C11—C10—C9	121.7 (8)	N5—O2—Nd1	95.6 (4)
C12—C11—C10	118.3 (10)	Nd1—O4—H4A	118.3
C12—C11—H11A	120.9	Nd1—O4—H4B	139.2
C10—C11—H11A	120.9	H4A—O4—H4B	97.6
C13—C12—C11	121.0 (9)	Nd1—O5—H5B	127.5
C13—C12—H12A	119.5	Nd1—O5—H5C	122.0
C11—C12—H12A	119.5	H5B—O5—H5C	107.0

Symmetry codes: (i) *x*, −*y*+1, *z*−1/2; (ii) −*x*+1, *y*, −*z*+1/2; (iii) *x*, −*y*+1, *z*+1/2.

### Hydrogen-bond geometry (Å, °)

**Table:**

*D*—H···*A*	*D*—H	H···*A*	*D*···*A*	*D*—H···*A*
O4—H4A···N4^iv^	0.85	2.00	2.760 (9)	149.1
O4—H4B···N3^v^	0.85	2.00	2.814 (10)	160.5
O5—H5B···N9^vi^	0.85	2.16	2.993 (9)	167.4
O5—H5C···O1^i^	0.85	1.99	2.770 (8)	152.2

Symmetry codes: (iv) −*x*+1/2, *y*−1/2, −*z*−1/2; (v) −*x*+1/2, −*y*+3/2, −*z*; (vi) −*x*+1, −*y*+1, −*z*; (i) *x*, −*y*+1, *z*−1/2.
